# Three-Dimensional Computer-Aided Design of a Full-Color Ocular Prosthesis with Textured Iris and Sclera Manufactured in One Single Print Job

**DOI:** 10.1089/3dp.2021.0048

**Published:** 2021-12-09

**Authors:** Annabel L.W. Groot, Jelmer S. Remmers, Dyonne T. Hartong

**Affiliations:** Department of Ophthalmology, Amsterdam UMC, University of Amsterdam, Amsterdam Orbital Center, Amsterdam, Netherlands.

**Keywords:** ocular prosthetics, eye prosthesis, 3D printing, innovations, displacement mapping, computer-aided design, and computer-aided manufacturing

## Abstract

Three-dimensional (3D) printing of ocular prosthesis has been scarcely described in medical literature. Although ocular prostheses have been 3D printed successfully, iris colors are often manually added to the final product afterward. The objective was to produce a 3D-printed ocular prosthesis with textured iris and sclera in one single print job. We designed an average 3D model of an ocular prosthesis in 3D software, and took a high-resolution digital photograph of a human eye, which was processed in graphical software. By using functions called “displacement mapping” and “UV mapping” on the 3D model, the extent of height displacement was used to digitally produce a textured and colored iris and sclera on the 3D model. By using a polyjet 3D printer, different colors and materials could be used for different prosthesis components. We were able to design and 3D print a lifelike ocular prosthesis with realistic iris and sclera texture. The process took less than 4 h, of which 2.5 h are “printing time,” reducing labor time compared with conventional methods. This proof-of-concept adds valuable knowledge to the future manufacture of 3D-printed ocular prostheses, which has several benefits over the conventional production method: 3D printing is much faster, reproducible, and prostheses can easily be digitally adjusted and reprinted. This study is an important step in the development of a full-fledged 3D workflow to produce lifelike custom eye prostheses.


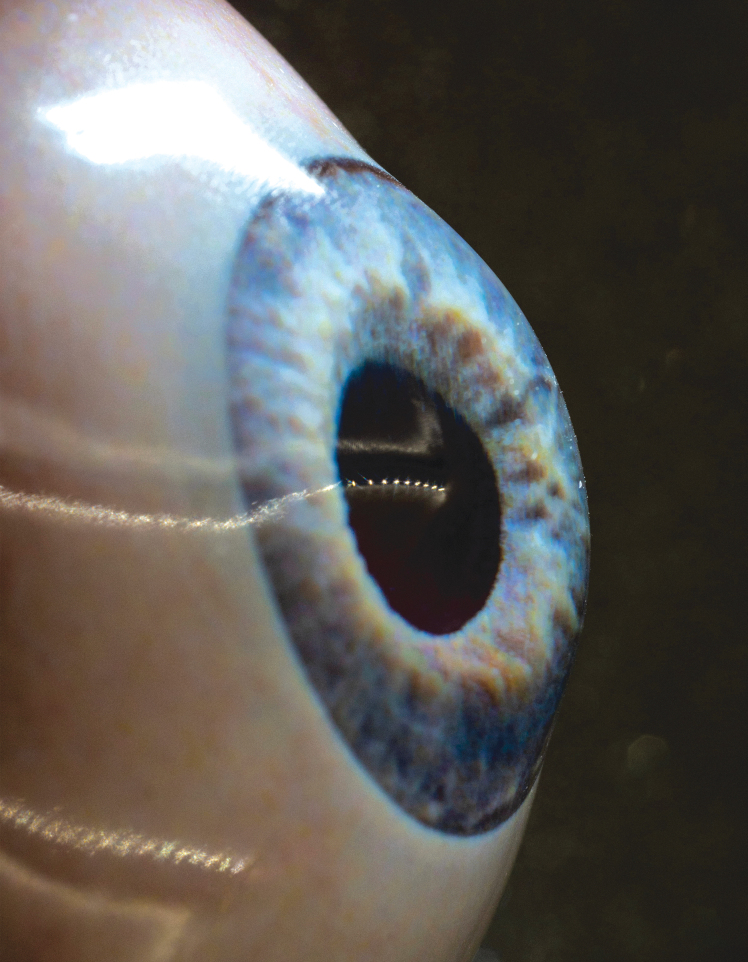


## Introduction

Fabricating artificial eyes is currently a delicate process performed by an ocularist, using techniques that have been refined over the past hundreds of years. Such an ocular prosthesis should ideally match the unaffected eye in shape and color. The lifelike reproduction of a human iris with its pigmented, layered texture with crypts and folds is particularly challenging.^1^ These detailed properties are difficult to capture by conventional production of a polymethyl methacrylate prosthesis, where the iris is made by applying paint in several semitransparent layers. Although a well-skilled professional will achieve satisfying results, the manual production of an ocular prosthesis is both time-consuming and nonreproducible.

Three-dimensional (3D) printing provides a reproducible method to create custom-made objects for various purposes.^2^ Methods for digitally capturing ophthalmic structures have also been described by Disney for graphical applications in their films and computer games.^3–6^ Inspired by these articles, we propose this proof-of-concept for manufacturing a realistic, 3D-printed ocular prosthesis with lifelike colors and texture in one single print job.

## Materials and Methods

As the research involved the researchers as test subject only, approval from the ethics committee was waived. A flowchart for the process is seen in Table 1.

**Table 1.** Flowchart for Three-Dimensional Design of an Ocular Prosthesis

(1) Design a basic prosthesis with core (iris and sclera) and external geometry (anterior chamber and cornea) and save as .stl in Meshmixer
(2) Take high-resolution photograph of eye with clearly visible iris
(3) Postprocess in Adobe Photoshop, save full-color and grayscale versions of the image as .png
(4) Load core geometry (.stl) and grayscale (.png) into Blender, select the iris on the core geometry and use “displacement mapping,” creating a textured iris
(5) Use “UV editing” in Blender to copy the full-color image onto the selected iris on the core geometry
(6) Repeat for scleral veins and save as .obj
(7) Import core geometry (.obj) and exterior geometry (.stl) into 3D printer software and assign color and resin
(8) Print, remove support material, and manual polish afterward

3D, three dimensional.

**FIG. 1.**
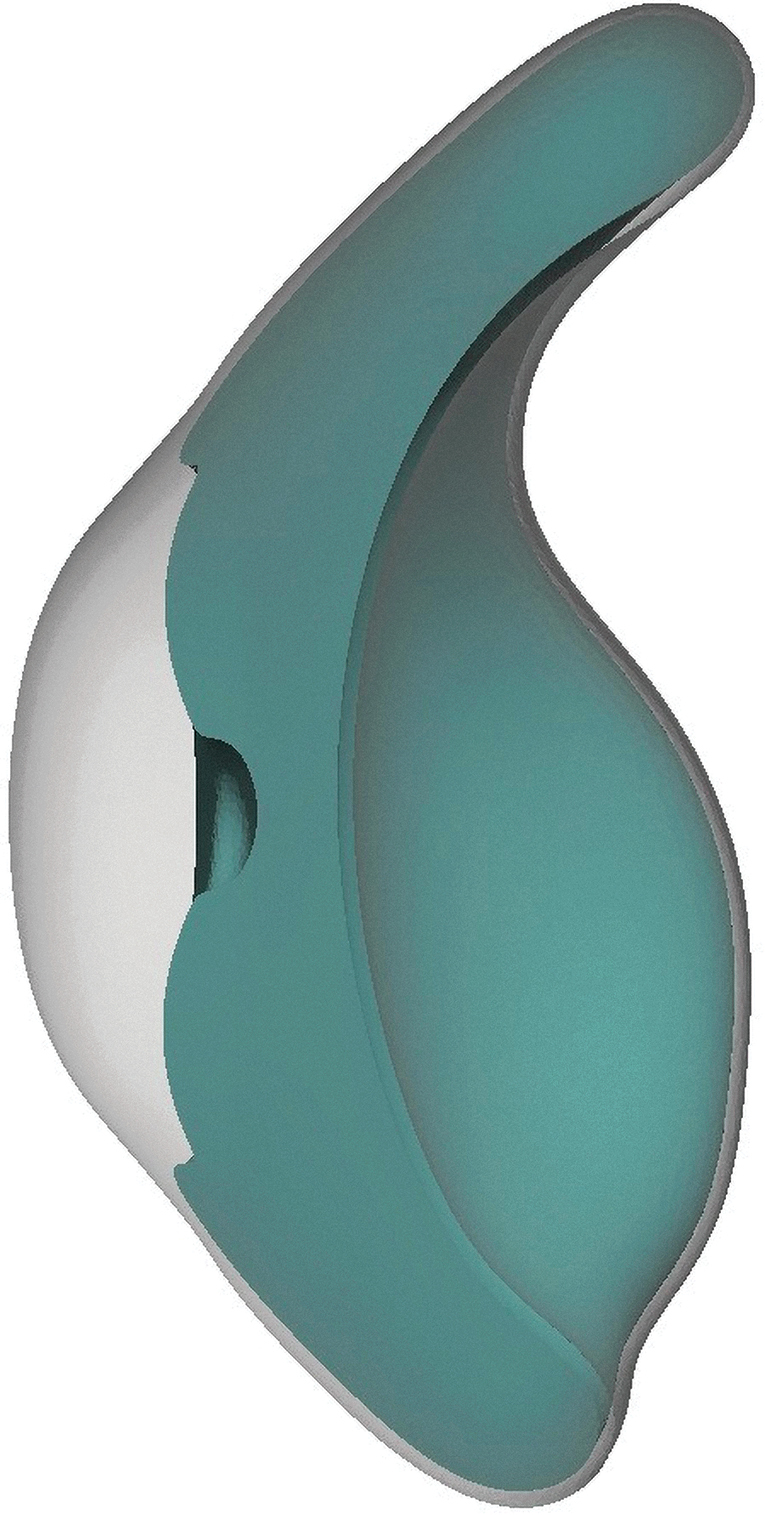
.stl file from Meshmixer showing a cross section of the prosthesis model.

We created an average-shaped prosthesis model and saved it as .stl file in Meshmixer (Autodesk, Inc., San Rafael, CA) consisting of the following: (1) the colored part of the eye or the iris; (2) the white part of the eye or the sclera (these two combined are referred to as “core geometry”); and (3) transparent anterior chamber and cornea (“transparent exterior geometry”). We designed the core geometry as one model with a cross-sectioned donut shape with a height of 2 mm and a diameter of 12 mm for the iris with a 3 mm central hole for the pupil, reflecting average human anatomy. The exterior transparent geometry had a maximum thickness of 3 mm at the central cornea created as a dome shape and merged with a 0.5 mm transparent layer covering the complete core geometry (Fig. 1). If available, a personalized prosthesis model can also be used instead of using an average-shaped prosthesis.

**FIG. 2.**
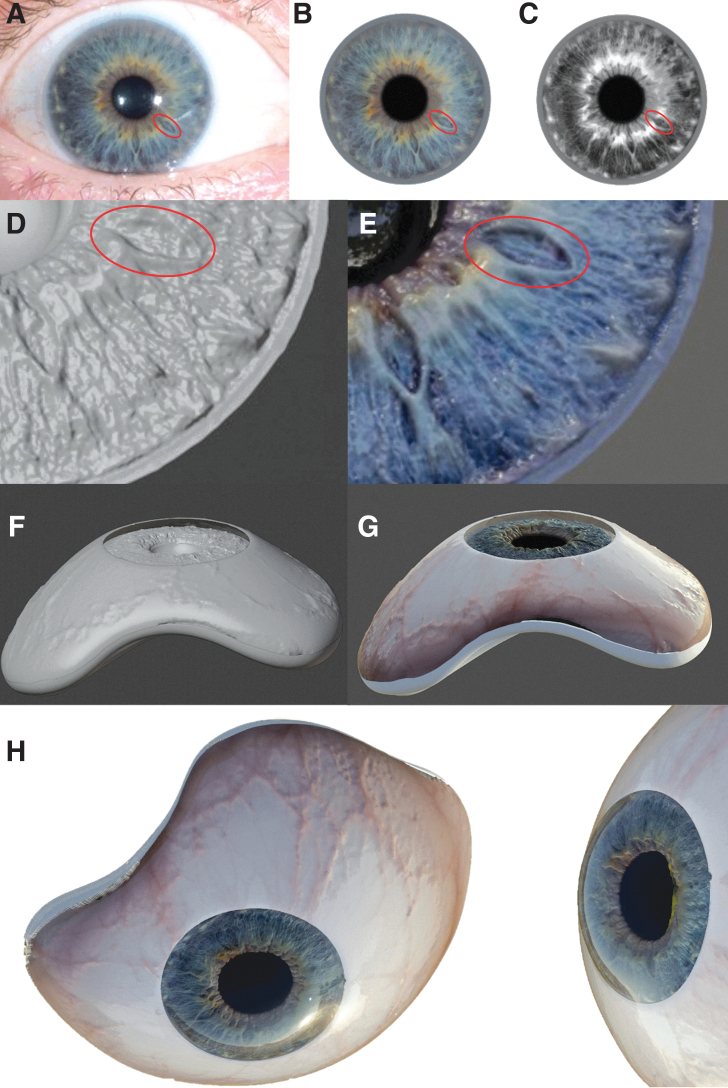
3D design of a lifelike, textured, and full-color iris. **(A)** The high-resolution digital photograph. **(B)** The iris is isolated from the photograph using Adobe Photoshop and postprocessed. **(C)** Duplicate grayscale image. **(D)** The displacement mapping function in the 3D-design program Blender creates height displacement and therefore a texture on the selected iris of the core geometry. **(E)** UV mapping the colored image onto the texture creates a full-color and textured 3D iris; **(F)** The procedure was repeated for the sclera. **(G)** Core geometry with textured and colored iris and sclera. **(H)** Rendered, photorealistic image of the (full-color) core and (transparent) exterior geometry combined, showing the result. The final prosthesis model is now ready to print. 3D, three dimensional.

We then took a high-resolution photograph of one of the researcher's eyes (JSR) from ∼30 cm using a gray card for white balance with a professional digital camera (Olympus E-M10 Mark II with macro lens 60 mm f/2.8, settings ISO100 f/13 1/160 s, RAW export, 4608 × 3456px). In Adobe Photoshop 2018 (Adobe Systems, Inc., San Jose, CA), the image was postprocessed: this includes calibrating colors using the gray card, covering missing parts and artifacts, and adjusting the iris shape into a circle with a centered pupil. A duplicate grayscale image served as an “elevation map,” where darker shades represented the iris crypts (thus being “deeper” or “more posterior”) and lighter shades represented more protruding iris parts (thus being “higher” or “more anterior”). This image was loaded in 3D-design software (Blender, Amsterdam, the Netherlands) where the function “displacement mapping” determines pixel values in the grayscale image. We also imported the .stl prosthesis file from Meshmixer into Blender, selected the (still smooth) iris in the “core geometry” and applied the “displacement mapping” function. In this way, points on the iris were displaced in positive or negative direction depending on the corresponding grayscale values, and so, the extent of height displacement was used to digitally produce a 3D textured iris. The colored version of the photograph (.png) was subsequently mapped onto this surface using the function “UV editing” in Blender, creating a full-color and textured iris. The process was repeated for the sclera to produce realistic color and veins. The core geometry with color and texture was exported as .obj file. Hereafter, the transparent exterior was added in Blender to create a glossy eye with natural depth and exported as .stl file (Fig. 2).

Finally, these files were loaded in accompanying software for the Stratasys J750 PolyJet 3D-printer where different resins or colors can be assigned to the different parts of the model (GrabCAD PRINT, StrataSys Ltd., Israel for Stratasys J750 printer). The “core geometry” was printed using Stratasys VeroBlackPlus, VeroCyan-V, VeroClear, VeroMagenta-V, VeroPureWhite, Vero-Yellow-V, and FullCure705. The “exterior geometry” was printed with ultraclear resin (Stratasys VeroUltraClear). Since the ultraclear resin is not biocompatible, we added another 0.2 mm of biocompatible resin as a coating to create a biocompatible model (Stratasys Biocompatible Clear MED 610). After printing, the prosthesis was polished using pumice stone powder and a dental rag wheel, according to standard procedures in ocularist practices.

## Results

**FIG. 3.**
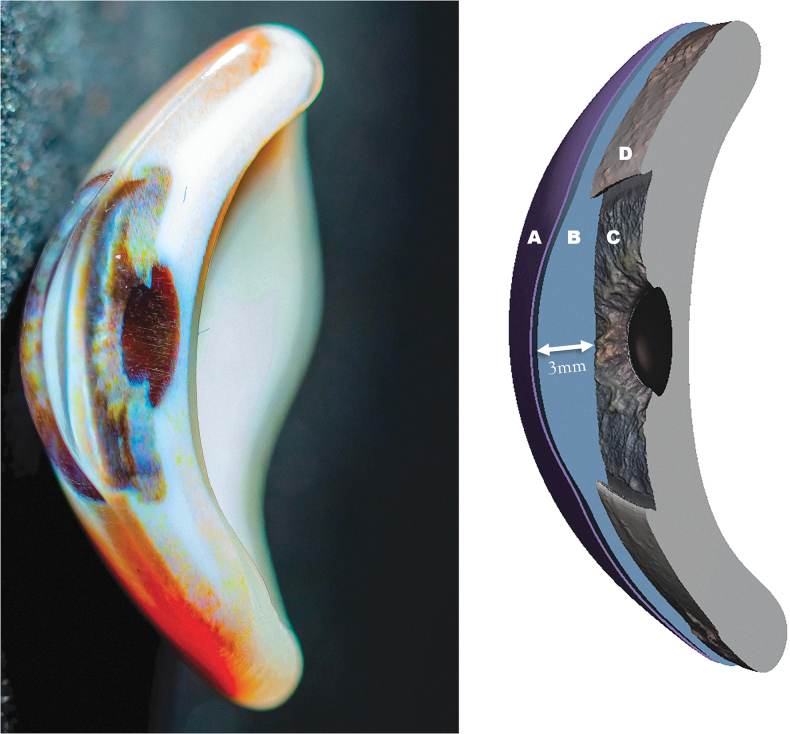
*Left*: a digital photograph (cross section) of the 3D-printed ocular prosthesis, where the different layers, hollow pupil, and color can be clearly seen. *Right*: a schematic model where **(A, B)** make up the transparent exterior (cornea and anterior chamber with a maximum depth of 3 mm at the center of the model, as indicated by the *arrow*). **(C, D)** The core geometry (iris and sclera, respectively).

**FIG. 4.**
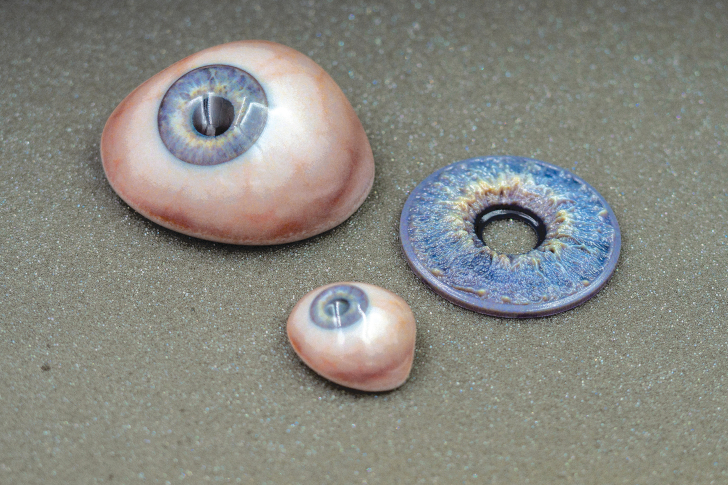
The final prosthesis (a large and normal size version) and a large version of the textured iris.

**FIG. 5.**
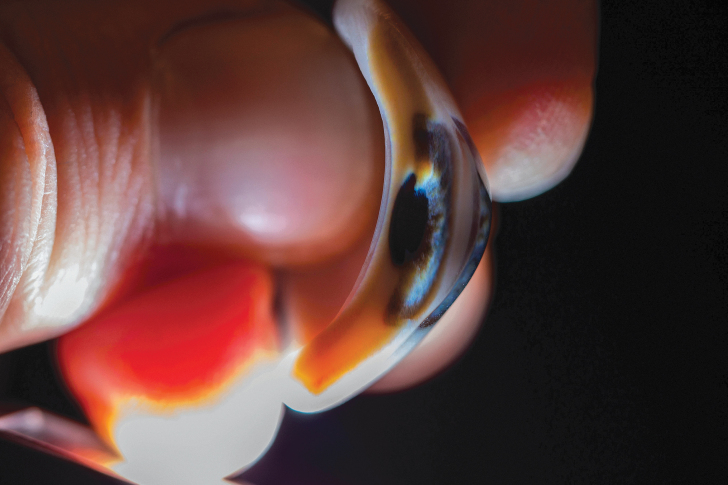
One of the researchers holding a cross section of the 3D-printed prosthesis.

A 3D-printed prosthesis with personalized iris and scleral color and texture was manufactured in a single print job (Figs. 3–5). The design and printing process took less than 4 h in total, in contrast to the 10 h of conventional manufacturing: 1 h of digital preparation, 2.5 h for printing, and 20 min for polishing. The clear cornea and anterior chamber give depth to the prosthesis and facilitate a clear view on the iris structure, of which the color is transferred directly from the photograph. Shadows and highlights are casted through the folds and crypts so that color appears different depending on the incidence of light. Unlike a conventional prosthesis where the pupil is painted as a black circle, this pupil is an actual hole.

## Discussion

This proof-of-concept study shows that 3D printing of realistic eye anatomy with colored and textured iris and sclera in a single print job is possible. To the best of our knowledge, this method has not been described before. Medical literature regarding 3D printing of ocular prostheses is scarce. In 2013, a British company reported to be able to mass-produce 3D-printed prosthetic eyes with preset iris colors, leading to the possible production of 150 prostheses an hour.^7^ Belgian colleagues have investigated into defining prosthesis geometry with the use of an impression-free mold.^8,9^ In 2017, Alam described a 3D-printed prosthesis with hand-painted iris.^10^ Other experiments involving iris production include printing a photograph, coating it, and inserting it into the prosthesis, by painting a transparent contact lens and adding this to the prosthesis, and by printing an iris photograph on transfer paper and adding it to the prosthesis by heat.^11–13^ In this study, we investigated into producing realistic and personalized eye prostheses, contrary to the existing companies selling cheaper prosthetic eyes for taxidermy purposes or mass-produced prosthetic eyes that often lack exact color-matching to the contralateral eye, which is important for patients wearing their prosthesis in daily life.

A big advantage over conventional production methods is that 3D printing is reproducible. A new prosthesis is obtained more quickly if a patient loses his or her prosthesis, small modifications can be easily done digitally, and a prosthesis could then theoretically be manufactured without the patient even visiting the ocularist's office. The most important advantage is saving time, as the conventional production is ∼10 h.^10,13^ We produced this single model in less than 4 h, of which 1 h is digital preparation, 2.5 h are “printing time” and can therefore be used to do other work, and 20 min are polishing. In an optimal process, the printer's tray is full with 154 pieces resulting in a printing time of 11 min per prosthesis. Obviously, some knowledge of 3D software and experience with 3D-design computer programs speed up the process. Future developments of 3D printing will probably enhance the quality and increase the speed of the production process as well.

A current challenge remains to obtain an iris color that most closely resembles the natural eye. Technically, accurate color reproduction is difficult as a 3D printer is still limited in its number of colors and the colored materials are highly translucent. This translucency leads to different perception of color depending on the underlying colors, light settings, and the primary digital photograph.^3^ This experiment is also still proof-of-concept, as the purchase of a polyjet 3D printer does currently not outweigh the benefits of 3D printing ocular prostheses, especially for a low amount of prostheses. Sharing a printer in larger health care institutions or bundling production for larger countries may be a cost-effective option.

The used resin is suitable for 30 days of skin contact and up to 24 h of mucosal membrane contact. For clinical application, further cytotoxicity studies and a certification procedure are essential. Experiments with a thicker layer of MED610, which would permit us to omit the “VeroUltraClear” resin, resulted in an opaque cornea appearance and poor visibility of the iris. Properties such as color stability, biocompatibility, and material longevity will need further investigation before clinical application is possible. Nevertheless, we believe this proof-of-concept is an important step in the development of a full-fledged 3D workflow to produce lifelike custom eye prostheses.

## Conclusions

By developing a novel method of adjusting a digital photograph of a human eye, this proof-of-concept adds valuable knowledge to the future manufacture of 3D-printed ocular prostheses, which has several benefits over the conventional method.

## References

[1] Eagle RC Jr. Iris pigmentation and pigmented lesions: An ultrastructural study. Trans Am Ophthalmol Soc 1988;86:581–687. 2979031 PMC1298824

[2] Hoang D, Perrault D, Stevanovic M, *et al.* Surgical applications of three-dimensional printing: A review of the current literature & how to get started. Ann Transl Med 2016;4:456. 28090512 10.21037/atm.2016.12.18 PMC5220021

[3] Brunton A, Arikan C, Urban P. Pushing the limits of 3D color printing: Error diffusion with translucent materials. ACM Trans Graph 2015;35:1–13.

[4] Bérard P, Bradley D, Nitti M, *et al.* High-quality capture of eyes. ACM Trans Graph 2014;33:1–12.

[5] Lefohn A, Caruso R, Reinhard E, *et al.* An ocularist's approach to human iris synthesis. IEEE Comp Graph Appl 2003;23:70–75.

[6] Chen H, Woodward MA, Burke DT, *et al.* Human iris three-dimensional imaging at micron resolution by a micro-plenoptic camera. Biomed Opt Express 2017;8:4514–4522. 29082081 10.1364/BOE.8.004514 PMC5654796

[7] Griffiths A. 3D printing “can produce up to 150 prosthetic eyes per hour” 2013 [Available from: https://www.dezeen.com/2013/11/26/3d-printed-prosthetic-eyes/

[8] Ruiters S, Sun Y, de Jong S, *et al.* Computer-aided design and three-dimensional printing in the manufacturing of an ocular prosthesis. Br J Ophthalmol 2016;100:879–881. 27121094 10.1136/bjophthalmol-2016-308399

[9] Ruiters S, Shujaat S, de Faria Vasconcelos K, *et al.* Three-dimensional design of a geometric model for an ocular prosthesis in ex vivo anophthalmic socket models. Acta Ophthalmol 2021;99:221–226. 32701212 10.1111/aos.14549

[10] Alam MS, Sugavaneswaran M, Arumaikkannu G, *et al.* An innovative method of ocular prosthesis fabrication by bio-CAD and rapid 3-D printing technology: A pilot study. Orbit 2017;36:223–227. 28375653 10.1080/01676830.2017.1287741

[11] Cevik P, Dilber E, Eraslan O. Different techniques in fabrication of ocular prosthesis. J Craniofac Surg 2012;23:1779–1781. 23147321 10.1097/SCS.0b013e31826701bb

[12] Buzayan MM, Ariffin YT, Yunus N, *et al.* Ocular defect rehabilitation using photography and digital imaging: A clinical report. J Prosthodont 2015;24:506–510. 25315047 10.1111/jopr.12235

[13] Ko J, Kim SH, Baek SW, *et al.* Semi-automated fabrication of customized ocular prosthesis with three-dimensional printing and sublimation transfer printing technology. Sci Rep 2019;9:2968. 30814585 10.1038/s41598-019-38992-y PMC6393501

